# UBQLN4 promotes progression of HCC via activating wnt-β-catenin pathway and is regulated by miR-370

**DOI:** 10.1186/s12935-019-1078-5

**Published:** 2020-01-03

**Authors:** Yan Yu, Penglin Xu, Guangying Cui, Xiaodong Xu, Kongfei Li, Xiaolong Chen, Jie Bao

**Affiliations:** 1 Precision Medicine Center, The First Affiliated Hospital of Zhengzhou University, Zhengzhou, 450052 China; 2 Key Laboratory of Clinical Medicine, Department of Digestive Diseases, The First Affiliated Hospital of Zhengzhou University, 1# Jianshe East Road, Zhengzhou, 450052 China; 3 Department of Hematology, Yinzhou People’s Hospital Affiliated to Medical College of Ningbo University, Ningbo, China

**Keywords:** Ubiquilin-4 (UBQLN4), Hepatocellular carcinoma (HCC), wnt-β-catenin pathway, MiR-370

## Abstract

**Background:**

Ubiquilin-4 (UBQLN4) is a member of the ubiquitin–proteasome system that is usually upregulated in many tumor cells. Its overexpression has been associated with poor disease outcomes in various cancer diseases. However, the underlying mechanism of UBQLN4 in the development of hepatocellular carcinoma (HCC) has not been elucidated.

**Methods:**

Immunochemistry, real-time PCR, and western blotting were used to evaluate the expression levels of UBQLN4 in cancer tissues. Univariate, Cox-regression, and Kaplan–Meier analyses were performed to determine the association between UBQLN4 expression and HCC prognosis. Cell Counting Kit-8 (CCK-8), transwell, EDU and colony formation assays were conducted to evaluate the role of UBQLN4 in HCC cell progression. The gene set enrichment analysis and luciferase reporter experiments were conducted to find the mechanism of UBQLN4 in HCC.

**Results:**

Ubiquilin-4 (UBQLN4) was overexpressed in HCC tissues. Besides, overexpression of UBQLN4 was associated with poor overall survival and disease-free survival rate of HCC patients. The loss-of-function analysis revealed that suppression of UBQLN4 inhibited the proliferation and invasion of HCC cells in vivo and in vitro. The KEGG (Kyoto Encyclopedia of Genes and Genomes) analysis showed that UBQLN4 could regulate activation of the wnt-β-catenin pathway in HCC cells. Furthermore, our results showed that UBQLN4 was downregulated by miR-370, which acted as a tumor suppressor gene in HCC progression.

**Conclusion:**

The results of the present study suggest that the miR-370/UBQLN4 axis may play a critical role in the progression of HCC. These findings may inform future strategies for the development of therapeutic agents against HCC.

## Background

Hepatocellular carcinoma (HCC) is one of the most common and highly malignant solid tumors, and is the third-leading cause of cancer-related deaths worldwide [1, 2]. Many treatment options for HCC exist, including surgical resection, liver transplantation (LT), transcatheter arterial chemoembolization (TACE), radiofrequency ablation (RFA) and molecular targeted therapy (sorafenib). These treatments can greatly improve the 5-year survival rate of HCC [3, 4]. However, because of the heterogeneity and high invasiveness of HCC cells, recurrence and metastasis are still common. Therefore, finding molecular markers for recurrence and metastasis in HCC is necessary for the improvement of survival rates of patients.

Ubiquilin protein (UBQLN) is an essential factor for the maintenance of protein homeostasis in cells. It acts as an adaptor protein that delivers ubiquitinated proteins to the proteasome [5]. There are five isomorphous proteins (UBQLN1–5) with a ubiquitin-like (UBL) domain at the N-terminus and a ubiquitin-associated domain (UBA) at the C-terminus. Members of the UBQLN family play an crucial role in maintaining homeostasis within the cell. Recent studies have demonstrated that UBQLN1 can inhibit apoptosis in lung cancer cells by stabilizing Bcl-B, and Bcl-2 family protein [6]; Ubiquilin can increase expression of p53 by affecting the degradation of p53 in a UBA domain-dependent manner [7, 8]. Ubiquilin-3 is overexpressed in the testis and regulates cell cycle progression during spermatogenesis [9]. Also, many studies have revealed the vital role of UBQLNs in the progression of various human cancers. Ubiquilin -2 is involved in migration and invasion of breast cancer [10] and lung cancer cells [11]. A recent study showed that UBQLN4 could curtail HRR activity by removing MRE11 from damaged chromatin and provide a therapeutic target for PARP1 inhibitor treatment in UBQLN4-overexpressing tumors [12]. Ubiquilin -4 acts as a tumor suppressor during the progression of gastric cancer by inducing cell cycle arrest and senescence via the extracellular signal-regulated kinases (ERK) signaling pathway [13, 14]. However, the function of UBQLN4 in the initiation and progression of HCC is still unclear.

In this study, UBQLN4 was overexpressed in HCC tissues, and this was associated with poor prognosis of HCC patients. Suppression of UBQLN4 inhibited tumor formation, proliferation, and invasion in vitro and in vivo by regulating wnt-β-catenin pathway. Moreover, UBQLN4 was the downstream target of miR-370. Overexpression of UBQLN4 reversed tumor suppressive function of miR-370. Collectively, the results of the present study show that miR-370/UBQLN4 axis regulates the formation and progression of HCC. These findings may provide a potential target in the development of therapeutic strategies against HCC.

## Materials and methods

### Patient samples and tissue microarray

Pan-cancer tissue microarray (Pan-cancer TMA) contained ten solid tumors (each tumor contained 20 pairs of tumor tissues and adjacent non-tumor tissues). Tissue microarray was performed on 341 HCC specimens, as previously described [15]. The tissues used in this study were acquired between January 2012 and December 2016 from the First Affiliated Hospital of Zhengzhou University, Zhengzhou University, China. Cancer Genome Atlas Project (TCGA; http://tcga-data.nci.nih.gov/) was used to analyze the relationship between UBQLN4 expression and development of solid tumors, according to the methods reported previously [16].

### Cell culture

Normal liver cells (Chang Liver and L02) and HCC cell lines (HepG2, SMMC-7721, SK-Hep-3B, HCC-LM3 and MHCC-97H) were procured from Sibcb (Shanghai, China). Chang Liver and L02 were cultured in 1640 supplied with 10% fetal bovine serum (FBS), whereas HCC cells were cultured in MEM supplied with 10% fetal bovine serum. The cells were incubated at 37 °C in a humidified atmosphere of 5% CO_2_.

### Transfection of cell lines

Human-specific shUBQLN4 (1–3) and negative control vectors were used to develop stable UBQLN4 suppression cell lines. Human UBQLN4 overexpression plasmid and negative control were procured from Gene Pharma (Shanghai, China). MiR-370 mimics, inhibitors, other miRNA mimics, and the controls (NC) were procured from Gene Pharma (Shanghai, China). The cell transfection procedure was conducted according to the manufacturers’ instructions.

### Western blot and PCR assays

Protein isolation and western blotting were conducted as per the methods described previously [17]. Samples were probed with UBQLN4 (1:1000), β-catenin (1:1000), CyclinD1 (1:1000), Axin2 (1:1000), Wnt5a (1:1000), C-myc (1:1000), Actin (1:5000) and GAPDH (1:5000) monoclonal antibodies. Real-time polymerase chain reaction (RT-PCR) was conducted according to the methods described previously [18].

### Immunohistochemistry (IHC)

Pan-cancer Tissue microarray (Pan-cancer TMA) and TMA containing 341 HCC specimens were used to conduct immunohistochemistry analysis according to the methods described previously [19]. Also, immunohistochemistry analysis was conducted using tissues of nude mice, including sh-Ctrl, sh1-UBQLN4, and sh2-UBQLN4. The detection proteins included Ki67, UBQLN4, β-catenin, CyclinD1, Axin2, Wnt5a, and C-myc.

### Cell viability test, EDU assay, and Transmembrane invasion assay

Hep-G2 and SMMC-7721 cell lines were transfected with UBQLN4 shRNA1–2, and negative control (or miR-370 mimics, miR-370 mimics + UBQLN4 and negative control). Cell viability test, EDU assay and transmembrane invasion assay were conducted according to the methods described previously [20].

### Colony-forming and in vivo tumorigenesis assays

The SMMC-7721 cells transfected with shUBQLN4 or negative control were divided into two groups, one group was used for colony-forming assay in vitro, the other was used for subcutaneous tumor formation in nude mice. The number of clones in vitro, tumor volume and relative photon flux in vivo was detected and analyzed according to the methods described previously [16].

### The gene set enrichment analysis (GSEA)

The gene set enrichment analysis (GSEA) was conducted to evaluate correlated pathways based on UBQLN4 expression in HCC tissues. A total of 377 samples from TCGA data set were divided into two groups (UBQLN4 ^high^ and UBQLN4 ^low^). The specific bioinformatics analysis of GSEA v2.0 was conducted according to the methods described previously [15].

### Luciferase reporter and TOPFlash/FOPFlash reporter assays

Human Hep-G2 and SMMC-7721 cell lines were transfected with UBQLN4 shRNA1–2 and negative control (or miR-370 mimics, miR-370 mimics and UBQLN4 and negative control). Next, cells were co-transfected with the Wnt/β-catenin signaling reporter TOPFlash/FOPFlash (Addgene, Cambridge, MA, USA). The reporter-driven luciferase activity was quantified by analyzing the luminescence intensity using a Synergen 2 (BioTek) reader. The results are represented as normalized TOPFlash/FOPFlash values.

### Statistical analysis

All statistical analyses were performed using SPSS 18.0 software (SPSS Inc., Chicago, IL). A two-tailed paired Student’s t-test was used to analyze the variations between the two groups. The Chi square test was used to analyse differences in clinic-pathological factors between the CLK3 high- or low-expression patients. A *p < 0.05 was considered statistically significant.

## Results

### UBQLN4 expression is downregulated in cancer tissues

**Fig. 1:**
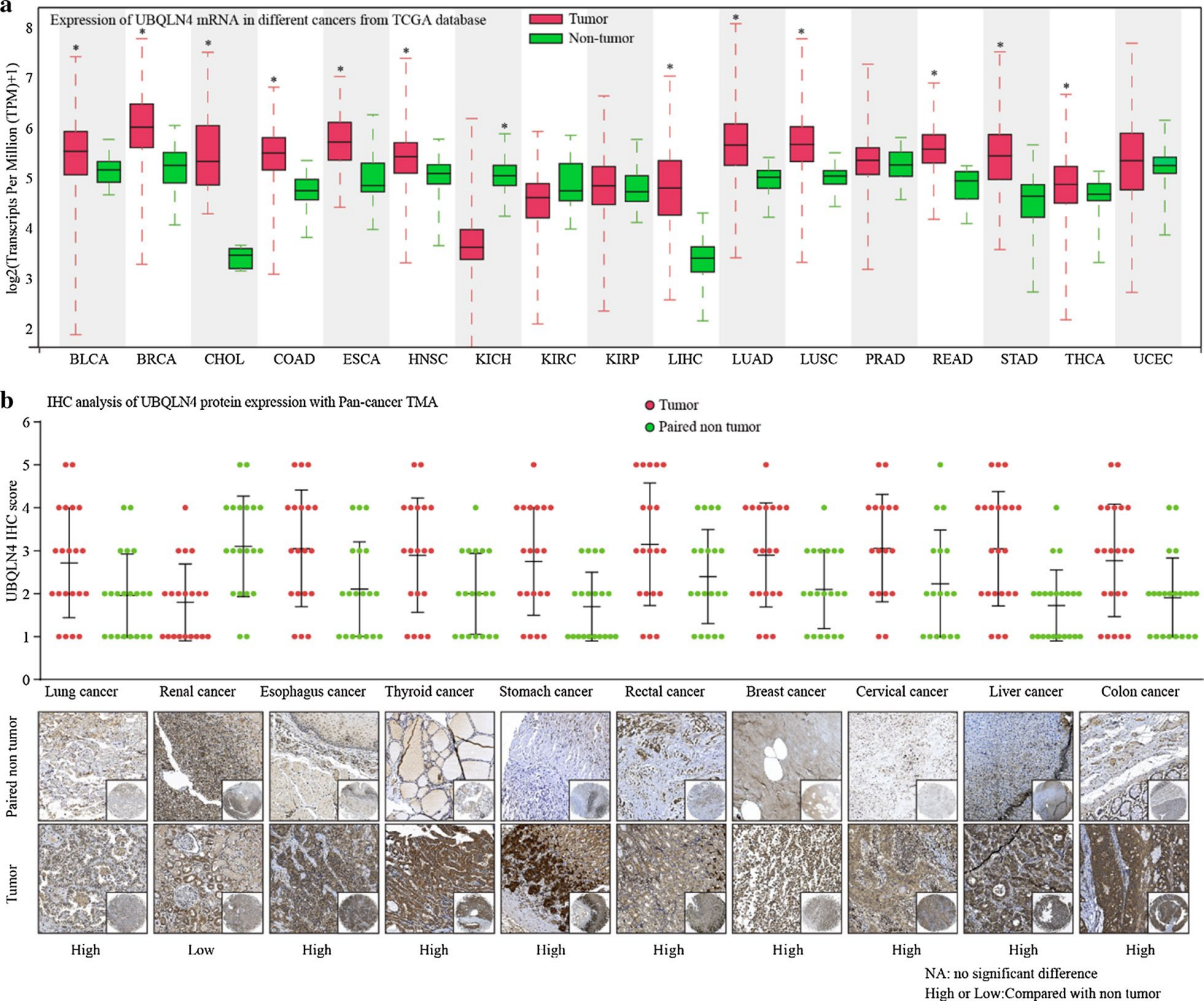
UBQLN4 is frequently dysregulated expression in cancers. **a** UBQLN4 mRNA expression level in TCGA data analysis. UBQLN4 mRNA was frequently overexpressed in most solid cancer tissues, in addition to KIRC, KIRP, PRAD and UCEC. **b** UBQLN4 protein expression in pan-cancer tissues and paired non-tumor samples was detected through IHC. By comparing the IHC score between pan-cancer tissues and paired non-tumor, UBQLN4 protein was upregulated in most solid tumors except for renal cancer. *p < 0.05, **p < 0.01

To investigate the expression of UBQLN4 in common solid cancers, the TCGA database was used to compare mRNA levels of UBQLN4 between tumor tissues and non-tumor tissues. The result showed that UBQLN4 mRNA levels were low in most solid cancer tissues, including KIRC, KIRP, PRAD and UCEC (Fig. 1a). Pan-cancer TMAs containing 21 pairs of cancer and normal adjacent tissues was performed to verify these results at the protein level. Ubiquilin-4 was overexpressed in most solid tumor tissues except in renal carcinoma (Fig. 1b). The increase in UBQLN4 expression suggested that UBQLN4 might play an essential role in the initiation and progression of solid tumors, including HCC.

### Overexpression of UBQLN4 is correlated with poor prognosis of HCC patients

**Fig. 2:**
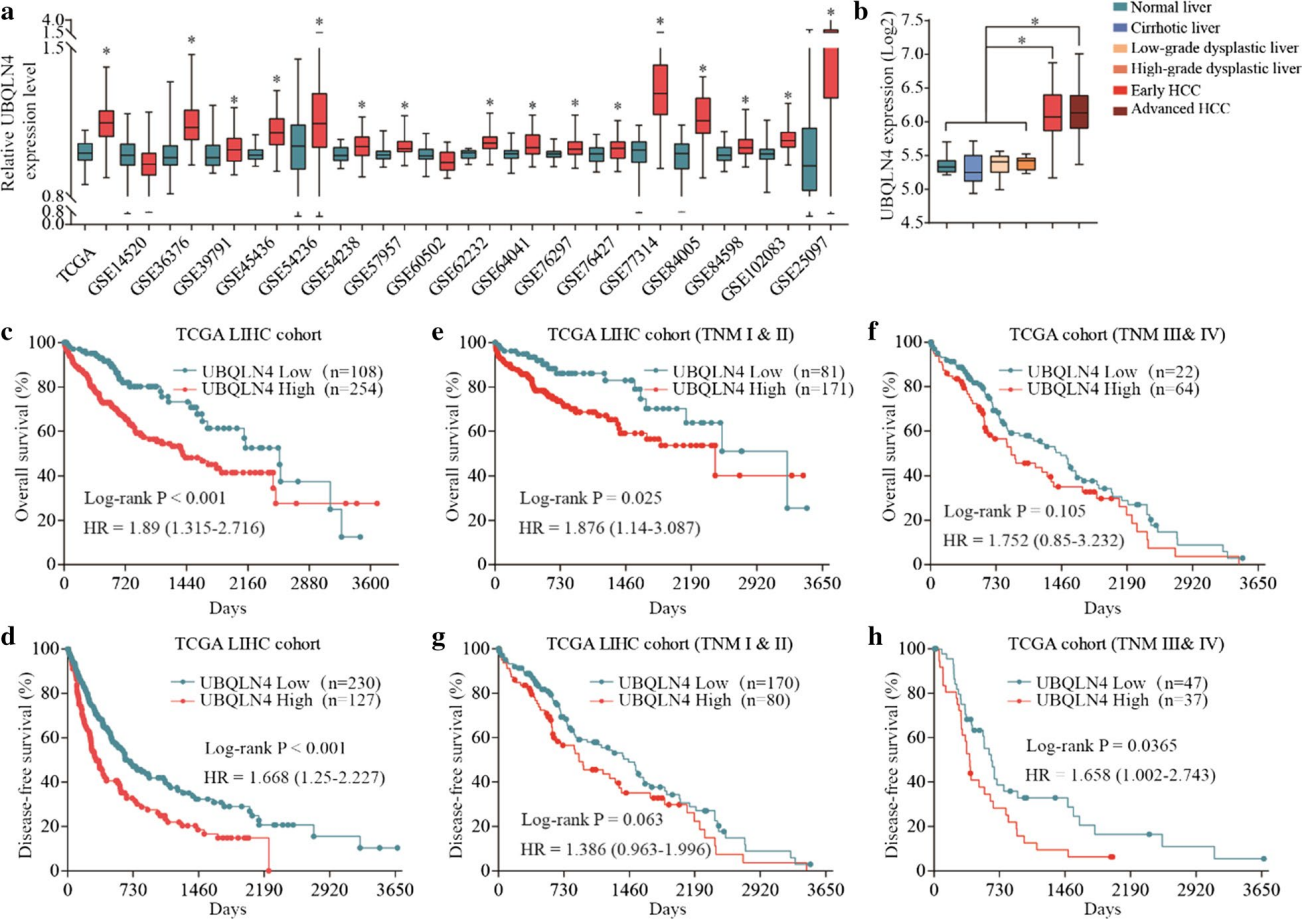
Overexpression of UBQLN4 is correlated with poor prognosis of HCC patients. **a** mRNA level of UBQLN4 in HCC tissues and normal tissues was analyzed through TCGA and GEA database. **b** Expression of UBQLN4 among normal liver, normal liver, cirrhosis liver, low-grade dysplastic liver, high-grade dysplastic liver, early HCC and advanced HCC was compared. **c**, **d** Kaplan–meier analysis was used to analyze relationship between UBQLN4 expression and prognosis of HCC, including overall survival and disease-free survival. **e**–**h** Stratified analyses between TNM stages and UBQLN4 expression on overall survival and disease-free survival. *p < 0.05, **p < 0.01

**Fig. 3:**
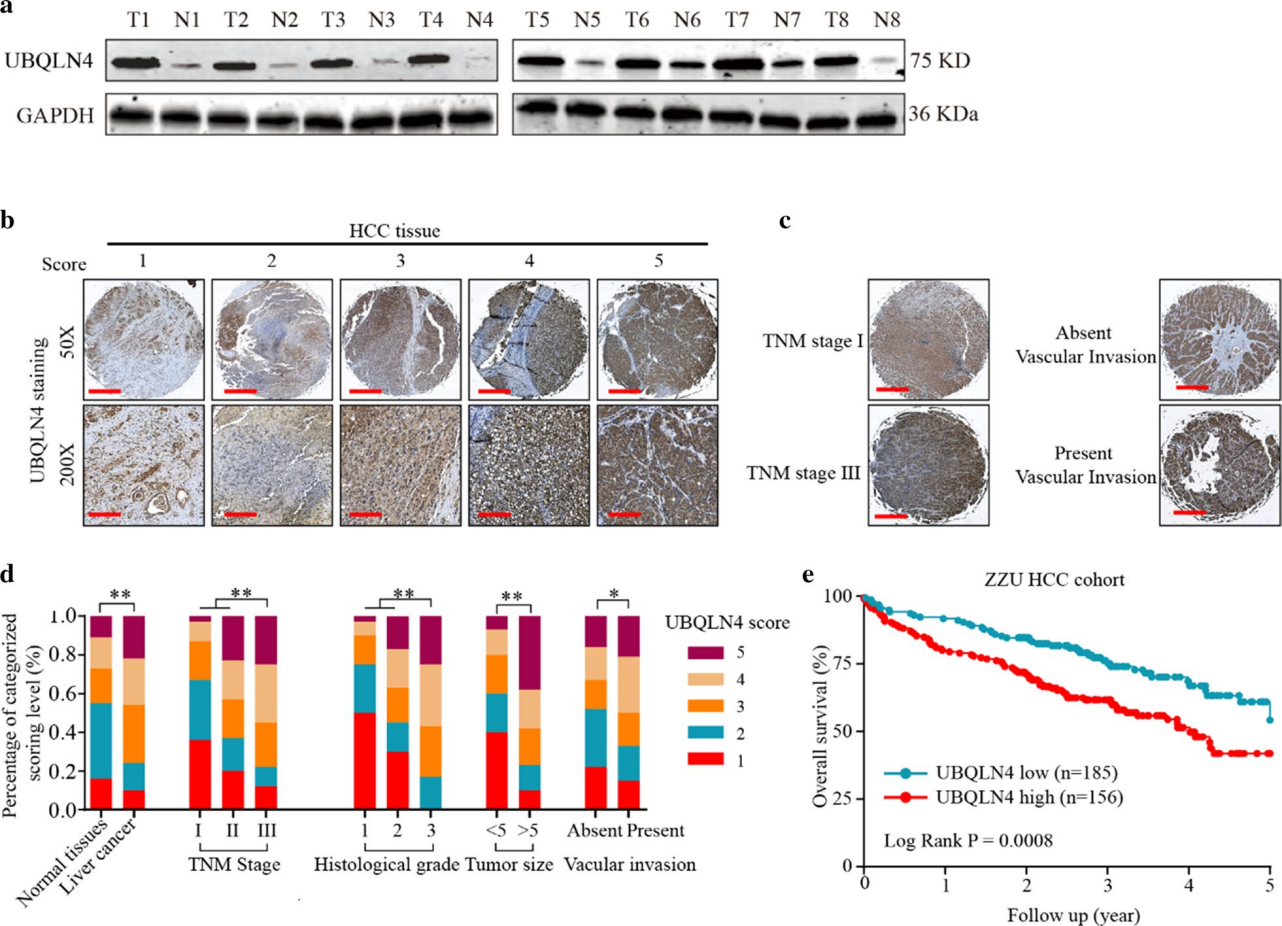
UBQLN4 is overexpressed in HCC tissues and associated with clinical pathological features of HCC patients. **a** Expression of UBQLN4 in HCC tissues and adjacent non-tumor tissues was detected by western blot. **b** HCC tissues from our hospital were detected by IHC and 1–5 points based on dyeing strength. **c**, **d** Relationship between expression of UBQLN4 and clinical pathological features of HCC patients (including TNM stage, poor histological grade, large tumor size and vascular invasion) was analyzed according to the IHC score. **e** Kaplan–meier analysis was used to analyze relationship between UBQLN4 expression and overall survival of HCC patients. *p < 0.05, **p < 0.01

To investigate the role of UBQLN4 in HCC progression, TCGA and GEO database analysis were performed to determine the variation in UBQLN4 mRNA levels between HCC and normal tissues. Expression of UBQLN4 was higher in most HCC tissues than normal tissues (Fig. 2a). Besides, UBQLN4 expression was higher in HCC tissues than in cirrhosis liver, low-grade dysplastic liver and high-grade dysplastic liver tissues (Fig. 2b). Kaplan–Meier analysis of TCGA LIHC cohort showed that high expression of UBQLN4 was correlated with poor overall survival and disease-free survival rates of HCC patients (Fig. 2c, d). Moreover, patients with high expression of UBQLN4 in TNM I-II group were associated with poor overall survival rate. However, there was no significant difference in overall survival time among the patients in TNM III-IV group (Fig. 2e, f). High expression of UBQLN4 observed in the TNM III-IV group was associated with poor disease-free survival; however, a similar observation was not made in TNM I-II group (Fig. 2g, h). To verify these results, UBQLN4 protein levels in 8 pairs of liver cancer and normal adjacent tissues were evaluated. Ubiquilin-4 was highly expressed in HCC tissues (Fig. 3a). Besides, high expression of UBQLN4 was associated with poor TNM stage, poor histological grade, large tumor size, vascular invasion and poor overall survival time (Fig. 3b–e) (Tables 1, 2). Collectively, these results showed that UBQLN4 is upregulated in HCC tissues and its expression is correlated with poor prognosis of HCC patients.

**Table 1:** Correlation of clinico-pathological features with UBQLN4 expression in ZZU HCC cohort

Variables	Clinicopathological features	UBQLN4 expression		*p-*value
		Low expression (n = 162)	High expression (n = 179)	
Age (years)	≤ 50	54	72	0.188
	> 50	108	107	
Gender	Male	125	140	0.816
	Female	37	39	
Pathogenesis	Virus	116	139	0.199
	Others	46	40	
Cirrhosis	Absent	147	168	0.279
	Present	15	11	
Tumor size (cm)	≤ 5	93	79	*0.014*
	> 5	100	69	
Vascular invasion	Absent	138	131	*0.006*
	Present	24	48	
TNM stage	Stage I and II	135	128	*0.009*
	Stage III and IV	27	51	
AFP	≤ 20	87	79	0.077
	> 20	75	100	
Tumor multiplicity	Single	87	72	*0.012*
	Multiple	75	107	
State	Live	120	110	*0.013*
	Dead	42	69	

Italics values indicate statistical significance, *p* < 0.05

**Table 2:** Correlation of clinico-pathological features with UBQLN4 expression in ZZU HCC cohort

	Univariate analysis			Multivariate analysis		
	HR	95% CI	*p* value	HR	95% CI	*p* value
Age (> 50 vs. ≤ 50)	1.143	0.884–1.413	0.863			
Gender (female vs. ≤ male)	0.846	0.534–1.342	0.479			
Pathogenesis (virus vs. ≤ others)	0.784	0.341–1.341	0.243			
AFP (> 20 vs. ≤ 20)	1.270	0.684–1.654	0.347			
Child-Pugh (C vs. A and B)	1.814	1.491–2.765	*0.029*	1.674	0.988–1.910	0.190
Cirrhosis (present vs. absent)	1.515	0.812–2.825	0.192			
Vascular invasion (present vs. absent)	1.848	1.225–2.793	*0.003*	2.180	1.416–2.404	*0.022*
TNM stage (III and IV vs. I and II)	3.333	2.262–4.902	*0.000*	3.907	2.613–5.461	*0.026*
Tumor size (> 5 vs. ≤ 5)	1.751	1.198–2.564	*0.004*	1.384	1.020–1.678	0.296
Tumor multiplicity (multiple vs. single)	2.964	2.176–3.472	*0.019*	1.977	1.323–2.401	0.054
UBQLN4 expression (high vs. low)	3.049	2.215–4.651	*0.024*	2.799	1.963–4.338	*0.006*

Italics values indicate statistical signiicance, *p* < 0.05

### Suppression of UBQLN4 inhibits proliferation and invasion ability of HCC cells in vitro

**Fig. 4:**
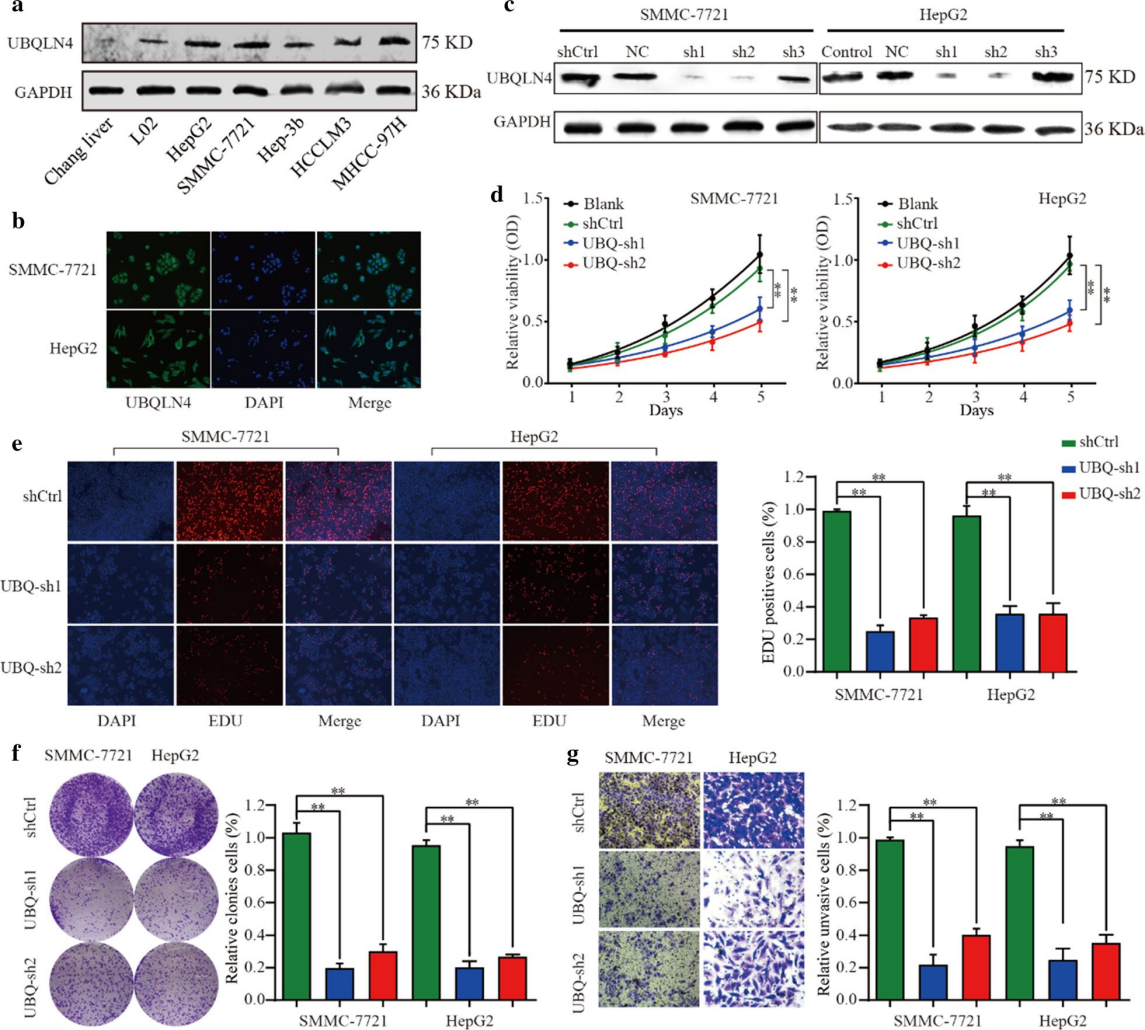
Suppression of UBQLN4 inhibits proliferation and invasion of HCC cells in vitro. **a** Protein level of UBQLN4 between normal hepatocytes cell lines and HCC cell lines was compared. **b** Expression of UBQLN4 in Hep-G2 and SMMC-7721 cells was detected through immunofluorescence. **c** Transfect efficiency of UBQLN4 shRNA in Hep-G2 and SMMC-7721 cells was verified in protein level. **d** Cell viability of Hep-G2 and SMMC-7721 cells under treatment of UBQLN4 shRNA and negative control in 24 h, 48 h and 72 h. **e**, **f** Cell proliferation rate and tumor formation ability of Hep-G2 and SMMC-7721 cells under treatment of UBQLN4 shRNA and negative control. **g** Invasion ability of HCC cells with UBQLN4 shRNA and negative control was compared in vitro. Mean ± SD, *p < 0.05, **p < 0.01

Evaluation of UBQLN4 expression in normal liver cell lines (Chang liver and LO2) and HCC cell lines (HepG2, SMMC-7721, SK-Hep-3B, HCC-LM3 and MHCC-97H) was conducted to investigate the role of UBQLN4 in HCC cell progression. Ubiquilin-4 was upregulated in HCC cell lines, especially in HepG2 and SMMC-7721 (Fig. 4a, b). Subsequently, cell lines were transfected with UBQLN4 knockdown vectors, and the knockdown efficiency was verified (Fig. 4c). Suppression of UBQLN4 inhibited the proliferation and clonal formation abilities of HCC cells (Fig. 4d–f). Also, UBQILN4 suppression reduced the cell invasion ability of HCC cells (Fig. 4g). Collectively, these findings revealed that UBQLN4 was crucial for HCC cell proliferation and invasion.

### Suppression of UBQLN4 inhibits tumor formation ability of HCC cells in vivo

**Fig. 5:**
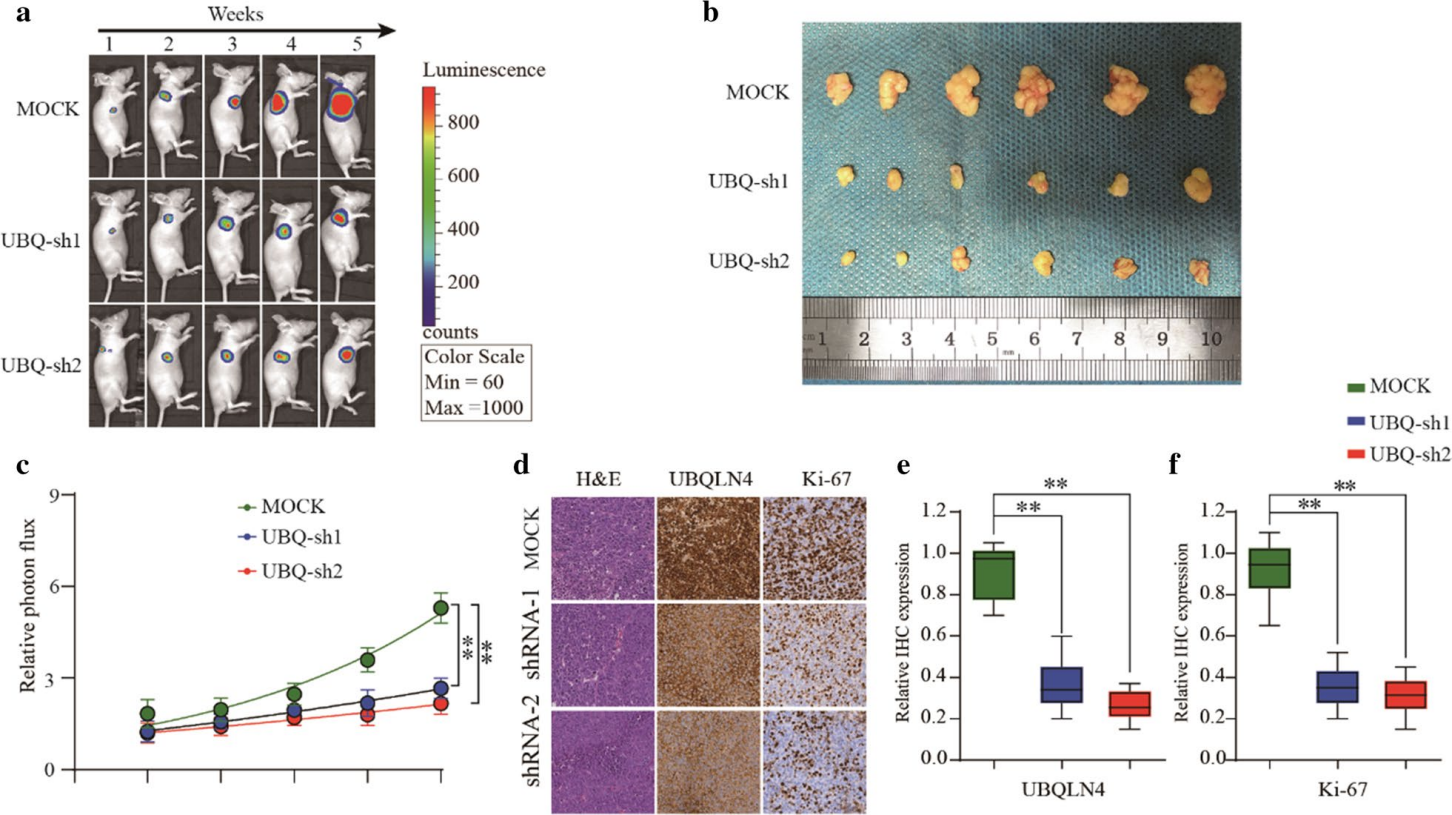
Suppression of UBQLN4 inhibits tumor formation ability of HCC cells in vivo. **a**–**c** The in vivo effect of UBQLN4 was evaluated in xenograft mouse models bearing tumors originating from SMMC-7721 cells via tumor volume and photon flux; n = 6 per group. **d**–**f** Expression of UBQLN4 and Ki67 were detected between UBQLN4 shRNA and negative control groups in node mouse tumor tissues. *p < 0.05, **p < 0.01

Subcutaneous tumor formation analysis in nude mice was conducted to further verify the role of UBQLN4 in HCC progression in vivo. The shUBQLN4 group had smaller tumor volume and weaker relative photon flux than the negative control group (Fig. 5a–c). Next, expression levels of UBQLN4 and Ki67 were evaluated in tumor tissues of nude mice. Ubiquilin-4 suppression led to low levels of UBQLN4 in shUBQLN4 group, and significantly reduced the expression of Ki67 (Fig. 5d–f). Thus, these results demonstrated that UBQLN4 suppression might inhibit tumor formation ability of HCC cells in vivo.

### UBQLN4 regulates proliferation and invasion of HCC cells through wnt-β-catenin pathway

**Fig. 6:**
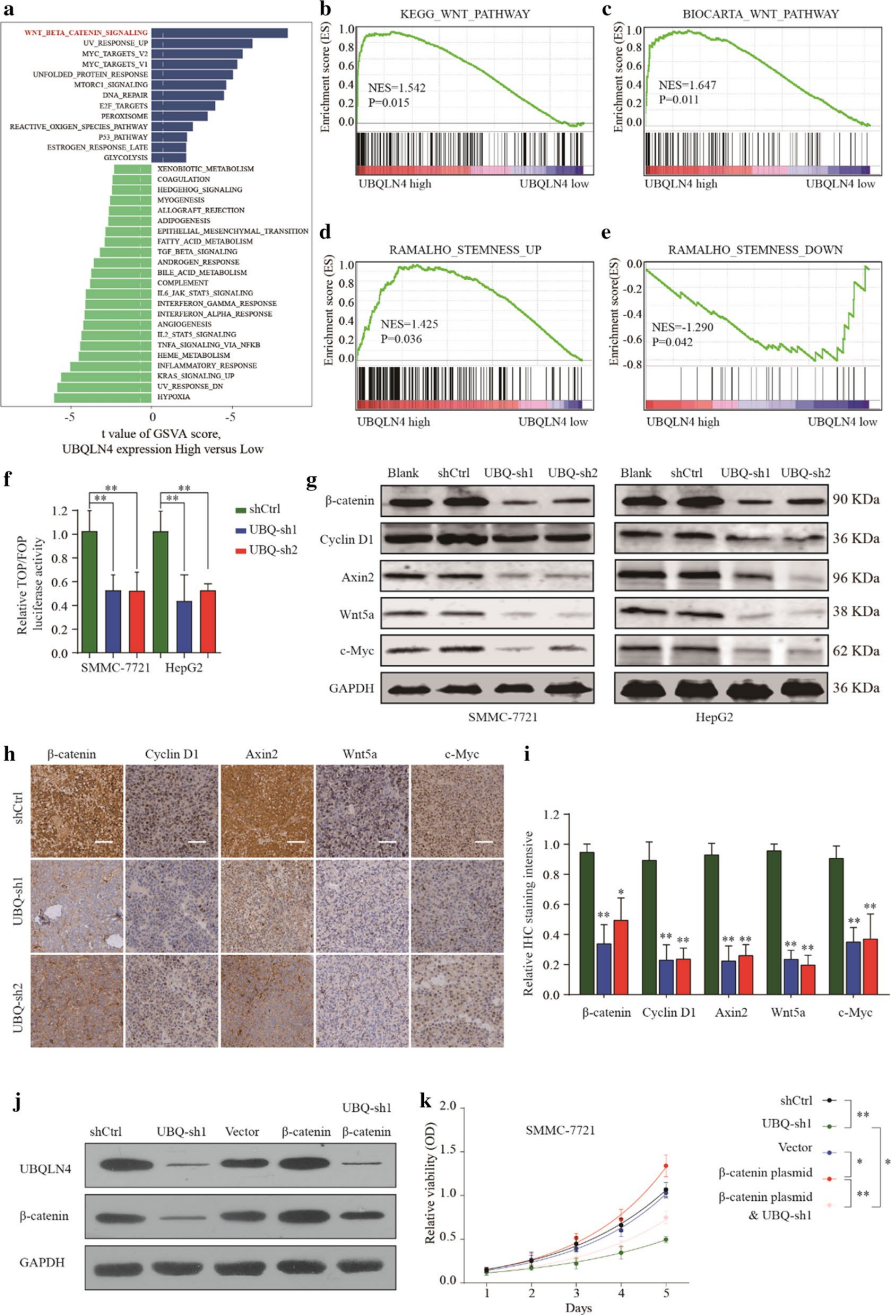
UBQLN4 regulates proliferation and invasion of HCC cells through wnt-β-catenin pathway. **a** The Gene Set Variation Analysis (GSVA) plot indicated a significant correlation between UBQLN4 and wnt-β-catenin pathway. **b**–**e** the Gene Set Enrichment Analysis (GSEA) showed the extensive positive correlation between UBQLN4 expression and Wnt pathway-related genes and stemness signature genes. **f** Dual luciferase assay demonstrating the effect on TOP/FOP reporter activity in Hep-G2 and SMMC-7721 cells transfected with shUBQLN4 and negative control. Results were normalized to a Renilla transfection control. **g**–**i** Expression change of β-catenin, CyclinD1, Axin2, Wnt5a and C-my in HCC cells and xenograft mouse models bearing tumors under condition of UBQLN4 suppression. **j** Showed UBQLN4 expression under different condition of negative control, shUBQLN4, vector plasmid, β-catenin plasmid or β-catenin plasmid and shUBQLN4. **k** Cell viability of SMMC-7721 cells under treatment of negative control, shUBQLN4, vector plasmid, β-catenin plasmid or β-catenin plasmid and shUBQLN4 in 24 h, 48 h and 72 h. *p < 0.05, **p < 0.01

A comprehensive bioinformatics analysis was performed based on TCGA database to investigate the mechanism underlying UBQLN4 roles in HCC progression. Gene Set Variation Analysis (GSVA) showed that UBQLN4 overexpression was correlated with the wnt-β-catenin pathway activation (Fig. 6a). Consistently, the Gene Set Enrichment Analysis (GSEA) further revealed the extensive positive correlation between UBQLN4 overexpression and Wnt pathway-related gene expressions (Fig. 6b, c). Wnt pathway and UBQLN4 expressions were significantly associated with stemness of solid tumors and signature genes, respectively (Fig. 6d, e). Furthermore, the results of the TOP/FOP experiment showed that UBQLN4 suppression significantly inhibited wnt-β-catenin pathway activation (Fig. 6f). Besides, suppression of UBQLN4 inhibited the expression of downstream genes involved in the wnt pathway, including β-catenin, CyclinD1, Axin2, Wnt5a and C-my (Fig. 6g–i). Mechanistically, UBQLN4 silencing impeded HCC cell growth, whereas reintroduction of β-catenin significantly reversed the inhibitory effects of UBQLN4 knockdown (Fig. 6j, k). Based on these findings, we verified that UBQLN4 might regulate proliferation and invasion abilities of HCC cells by affecting the activation of the wnt-β-catenin pathway.

### MiR-370 regulates UBQLN4 in HCC cells

**Fig. 7:**
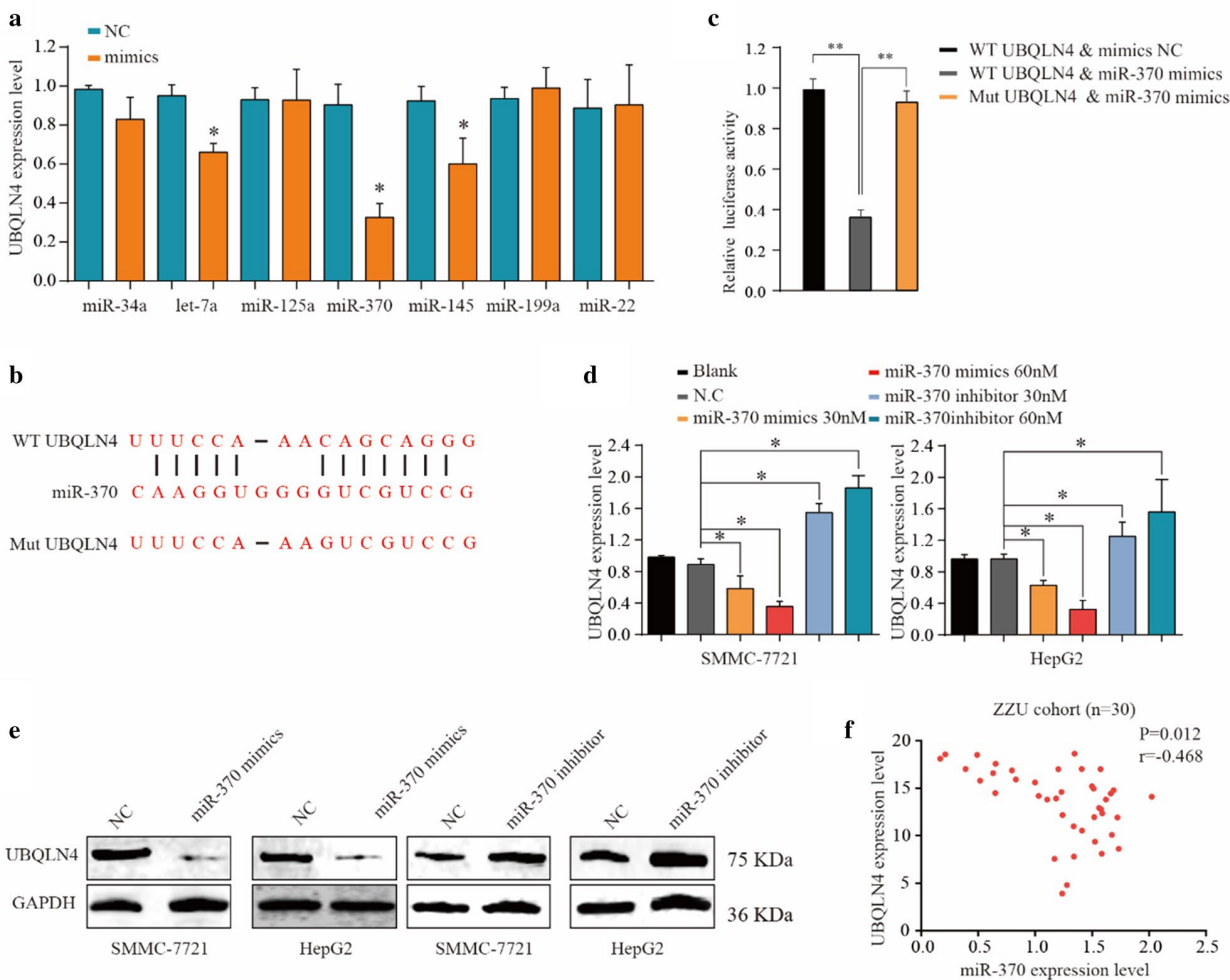
UBQLN4 was targeting regulated by miR-370 in HCC cells. **a** Expression of UBQLN4 was detected under treatment of selected miRNA mimics in HCC cells. **b** Relative luciferase activity between UBQLN4 wild type and mutant type based on a dual-luciferase reporter assay. Values represent the mean ± SD (n = 5, each). **c** miR-370 could be specific complementary pairing with 3′UTR sequence of UBQLN4. **d, e** Protein and mRNA change of UBQLN4 in HCC cells under treatment of miR-370 mimics, inhibitor and negative control. **f** Relationship between UBQLN4 expression and miR-370 expression in HCC tissues. *p < 0.05, **p < 0.01

Target Scan, miRTarBase, miRDB and miRcode were performed to investigate further the upstream regulatory gene of UBQLN4. Seven candidate miRNAs were complementary with 3′UTR of UBQLN4. Subsequently, miRNA mimics were used to suppress UBQLN4 expression in HCC cells. MiR-370 expression significantly reduced the UBQLN4 mRNA levels. Furthermore, luciferase experiment showed that miRNA-370 induced the degradation of UBQLN4 (Fig. 7a, b). MiR-370 mimics and inhibitors were co-cultivated with HCC cells to confirm the results further. MiR-370 mimics decreased mRNA and protein levels of UBQLN4 whereas miR-370 inhibitors enhanced UBQLN4 overexpression (Fig. 7c, d). Also, the expression of UBQLN4 was found to be negatively correlated with the miR-370 expression, as shown by TCGA database analysis (Fig. 7e).

### Overexpression of UBQLN4 could reverse tumor suppressive effect of miR-370 in HCC cells

**Fig. 8:**
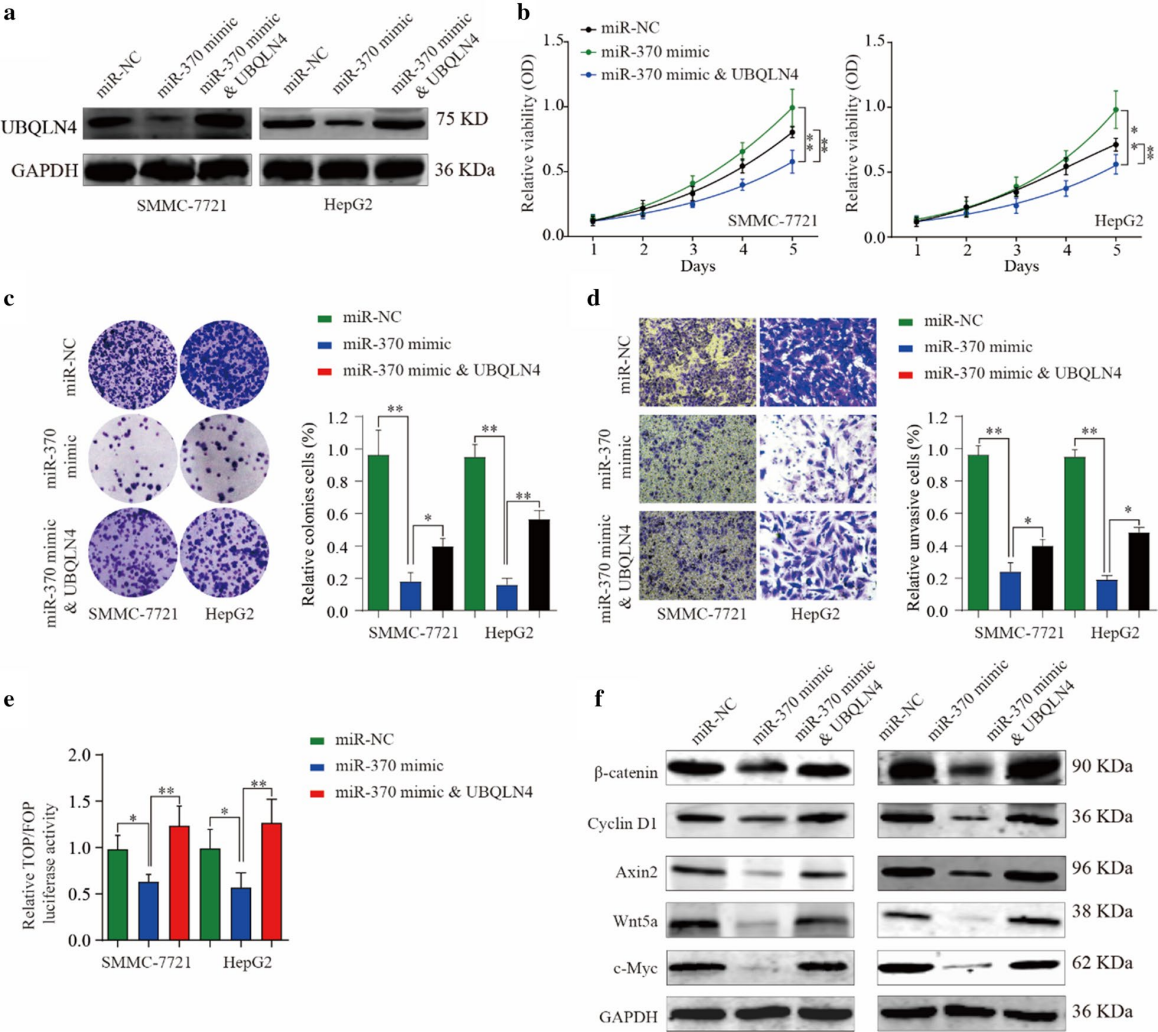
Overexpression of UBQLN4 could reverse tumor suppressive effect of miR-370 in HCC cells. **a** UBQLN4 expression under condition of miR-370 mimics, miR-370 mimics and UBQLN4 or negative control. **b** Cell viability of Hep-G2 and SMMC-7721 cells under treatment of miR-370 mimics, miR-370 mimics and UBQLN4 and negative control in 24 h, 48 h and 72 h. **c**, **d** Tumor formation ability and invasion ability of Hep-G2 and SMMC-7721 cells under treatment of miR-370 mimics, miR-370 mimics and UBQLN4 and negative control. **e** Dual luciferase assay demonstrating the effect on TOP/FOP reporter activity in Hep-G2 and SMMC-7721 cells transfected with miR-370 mimics, miR-370 mimics and UBQLN4 and negative control. Results were normalized to a Renilla transfection control. **f** Expression change of β-catenin, CyclinD1 and Axin2 in HCC cells under treatment of miR-370 mimics, miR-370 mimics and UBQLN4 and negative control. *p < 0.05, **p < 0.01

Ubiquilin-4 overexpression plasmid was used to verify further the relationship between UBQLN4 and miR-370 in HCC progression. The plasmid significantly reversed the inhibitory effect of miR-370 on UBQLN4 expression in HCC cells (Fig. 8a). Through the following cell function experiments, we observed that miR-370 expression inhibited the viability, clonal formation and invasion abilities of HCC cells, whereas overexpression of UBQLN4 partially reversed tumor suppressive functions of miR-370 (Fig. 8b–d). Moreover, results of TOP/FOP experiments showed that overexpression of UBQLN4 reduced the inhibitory effect of miR-370 on wnt signaling pathway. And this phenomenon was directly verified by a change in expression levels of wnt pathway associated genes, such as β-catenin, CyclinD1 and Axin2 (Fig. 8e). Collectively, the results of the present study showed that UBQLN4 overexpression could partially reverse the anti-tumor effects of miR-370 in HCC cells.

## Discussion

Hepatocellular carcinoma (HCC) accounts for 70–85% of primary liver cancers, which represents one of the most common fatal malignancies worldwide [21]. Despite improvements in surveillance and clinical treatment strategies, post-surgical recurrence and metastasis are still the main factors that limit the long-term survival of patients [22]. Because of limited number of predictive biomarkers for HCC recurrence and metastasis, it is necessary to identify new factors associated with tumor recurrence and understand the mechanisms underlying their role in HCC initiation and progression. The present study demonstrated for the first time that UBQLN4 is overexpressed in advanced HCC tissues, and high expression of UBQLN4 is associated with poor TNM stage, poor histological grade, large tumor size, vascular invasion and poor overall and disease-free survival rates. Loss of function experiments showed that suppression of UBQLN4 could significantly inhibit cell viability, proliferation rate, invasion ability and tumor formation ability in vitro and in vivo. Thus, UBQLN4 may be a novel prognostic molecular marker for prognosis and development of personalized therapy for HCC patients.

Bioinformatic analysis was performed to elucidate the downstream signaling pathway of UBQLN4, and the results revealed that activation of the wnt-β-catenin pathway was very crucial for the expression of UBQLN4. The wnt-signalling pathway has been associated with a wide range of cellular processes, and is activated by two cell surface receptors—the low-density lipoprotein receptor-related proteins 5 and 6 (LRP5/6) and frizzled [23–25]. Hyperactivation of the Wnt/β-catenin pathway can lead to aberrant cell growth and tumor invasion [26, 27]. Therefore, determining the mechanism of hyperactivation of this signaling pathway could be the key to finding new targets for tumor therapy. The results of the present study demonstrated that UBQLN4 suppression could significantly inhibit activation of the wnt-β-catenin pathway, and decrease expression of wnt-β-catenin pathway associated genes, including β-catenin, CyclinD1, Axin2, Wnt5a and C-myc. Besides, the effects of UBQLN4 silencing on cell proliferation in GBC cells was reversed by the reintroduction of β-catenin. This suggested that UBQLN4 might affect the progression of HCC cells by regulating the wnt-β-catenin pathway.

MicroRNAs (miRNAs) are a class of non-coding single-stranded RNA molecules of approximately 22 nucleotides in length. They bind specifically to complementary sites in the 3′ untranslated regions (3′UTRs) of mRNAs and cause mRNA degradation [28]. Many studies have demonstrated that miRNAs can affect relevant tumor progression pathways such as cell proliferation, apoptosis, and cell migration by regulating hundreds of target genes [29]. In this study, to further investigate upstream regulatory factors of UBQLN4 in the progression of HCC, we found multiple candidate miRNAs through bioinformatics analysis. Subsequently, confirmatory experiments were performed, which showed that miR-370 could specifically bind to the 3′UTR region of UBQLN4 and inhibit expression of UBQLN4 in HCC cells. By conducting cell function experiments, we observed that UBQLN4 overexpression reversed tumor suppressive roles of miR-370 in HCC cells. Furthermore, results of TOP/FOP experiments showed that overexpression of UBQLN4 reversed the inhibitory effect of miR-370 on wnt-β-catenin signaling pathway. Therefore, the findings of this study confirmed that UBQLN4 targets miR-370 (regulatory gene) to promote HCC progression.

## Conclusion

The results of the present study show that UBQLN4 is overexpressed in HCC tissues and acts as an independent risk factor for HCC prognosis. Suppression of UBQLN4 inhibits cell viability, proliferation, invasion and tumor formation abilities of HCC cells by suppressing the activation of the wnt-β-catenin signaling pathway. Furthermore, data showed that UBQLN4 is a downstream regulator gene of miR-370 in HCC. Collectively, these findings suggest that miR-370/UBQLN4/wnt-β-catenin axis plays an essential role in the progression of HCC and is a potential target for the development of therapeutic strategies against HCC.

## Data Availability

All the data and material could be traced from the paper we have published before.
